# Some Surgical Complications of Typhoid Fever

**Published:** 1908-01-18

**Authors:** 


### Points in Surgery.

### SOME SURGICAL COMPLICATIONS OF TYPHOID FEVER.

The case about to be described is of more than ordinary interest, not only on account of the unusual features which it presents, but also because it serves to lay stress on the fact that typhoid fever is a disease with many after effects and complications, of which a large proportion lie within the province of the surgeon rather than that of the physician. The patient, a man aged thirty-five, was attacked with typhoid fever. The disease ran an uneventful course, except for a prolonged period of delirium lasting three weeks. After a month the patient was pronounced to be out of danger, and convalescence began. At, or shortly after, this time he noticed a swelling in his lower abdomen, to which he called the attention of his medical man. It was not tender nor painful, except that it caused a dragging sensation in the abdomen if he lay on his left side. He asserted that it seemed to vary in size, being appreciably larger on some days than on others. The only other symptom complained of was frequent micturition; he was compelled to perform the act six times or more during the daytime, and several times at night. He had not, of course, been out of bed since the beginning of the attack.

On examination of the abdomen there was found to be a hard flat swelling below and to the right of the umbilicus. When the history of frequent micturition was taken into account, it was at first suggested that the swelling was in connection with the right ureter. But a more complete examination made it certain that the swelling was not intra-abdominal at all, but was in the right rectus abdominis muscle, or its sheath. Its lateral outlines corresponded with those of the muscle: on the inner side it extended to the middle line, and on the outer to the linea semilunaris. Above it reached nearly to the umbilicus, and below to within two inches of the symphysis. It moved with the movements of the abdominal wall. When the abdomen was fully relaxed the tips of the fingers could be insinuated underneath the tumour, and its posterior surface was felt to be quite flat. But when the patient made the abdomen tense by raising his head, the mobility of the tumour was immediately diminished and its outlines became indistinct. The examination caused him no pain.

The nature of this tumour forms an interesting problem. There can be no doubt, in view of the facts discovered on examination, that the tumour was in or connected with the rectus muscle; and, on account of the definite history, it is reasonable to presume that it was a direct result of the typhoid fever. The diagnosis seems to rest between some degenerative change in the muscle itself and hæmorrhage into its sheath subsequent to rupture of some of its fibres. There is a well recognised form of muscle degeneration following enteric fever which has been described by Zenker, and the fact that it attacks mainly the diaphragm and the recti abdominis may be said to be in favour of this diagnosis. Against it is the very definite outline of the tumour; for it must be supposed that if degeneration occurs the whole muscle is likely to be affected, and not a localised part of it. On the other hand, a hæmatoma into the rectus sheath which had clotted and commenced to organise would give rise to just such a tumour as the one described. Such hæmorrhage might be secondary to rupture of some of the fibres of the muscle. In this case no history could be obtained of any exertion likely to produce such a result. The delirium had been throughout of a low type, and restraint had never been necessary. But it may be argued that, if the muscle was degenerated at all, any slight exertion which would normally cause no damage, such as defæcation in the supine position, might be sufficient to cause rupture of some of the muscle fibres. In this case the problem must remain unsolved, because the tumour gave rise to no definite symptoms, so that an operation was not considered justifiable.

Typhoid fever is a disease complicated by some uncommon surgical complications, apart from general peritonitis following rupture of the intestine, which is such a well known danger of the disease.

Thrombosis may occur in almost any vein; when it does so it comes on during early or late convalescence. It is said most commonly to attack the left femoral vein, leading to œdema of that limb; but cases are certainly not uncommon in which the inferior vena cava is affected; and the writer has seen one case in which the superior vena cava was thrombosed. This complication retards convalescence since, apart from the constant danger of detachment of a thrombus and pulmonary embolism, it takes a long time for a collateral circulation to develop. In the case of thrombosis of the inferior vena cava this is effected by dilation of the superficial veins of the abdominal wall. Localised periostitis may occur during or after convalescence: most commonly it attacks the ribs, giving rise to a small circumscribed swelling. It may, however, attack any long bone. Clinically this form of periostitis seems to come mid-way between the ordinary acute and chronic surgical varieties; that is to say, the swelling is often found to contain pus, but the general constitutional symptoms of acute periostitis are absent. The bacillus typhosus can often be cultivated pure from this pus, a fact which proves that it is possessed of some chemiotactic properties. In other cases it is mixed with the bacillus coli or the ordinary pyogenic micro-organisms.

Cystitis of varying degrees of severity is also a frequent complication. Bacilluria can exist without cystitis; but there are cases in which the typhoid bacillus causes pyuria, and even gangrenous cystitis. Curiously enough, as in cystitis due to the bacillus coli, the pus is often found in acid urine. Acute cholecystitis has also been described, and it is said that the bacillus can be found in the gall bladder, in some cases years after the original infection.

